# Factors Affecting Microbial Load and Profile of Potential Pathogens and Food Spoilage Bacteria from Household Kitchen Tables

**DOI:** 10.1155/2016/3574149

**Published:** 2016-06-30

**Authors:** Susheela Biranjia-Hurdoyal, Melissa Cathleen Latouche

**Affiliations:** Department of Health Sciences, University of Mauritius, 80837 Reduit, Mauritius

## Abstract

The aim was to study the bacterial load and isolate potential pathogens and food spoilage bacteria from kitchen tables, including preparation tables and dining tables.* Methods*. A total of 53 households gave their consent for participation. The samples were collected by swabbing over an area of 5 cm by 5 cm of the tables and processed for bacterial count which was read as colony forming units (CFU), followed by isolation and identification of potential pathogens and food spoilage bacteria.* Result*. Knowledge about hygiene was not always put into practice. Coliforms,* Enterococcus* spp.,* Pseudomonas* spp.,* Proteus* spp., and* S. aureus* were detected from both dining and preparation tables. The mean CFU and presence of potential pathogens were significantly affected by the hygienic practices of the main food handler of the house, materials of kitchen tables, use of plastic covers, time of sample collection, use of multipurpose sponges/towels for cleaning, and the use of preparation tables as chopping boards (*p* < 0.05).* Conclusion*. Kitchen tables could be very important source of potential pathogens and food spoilage bacteria causing foodborne diseases. Lack of hygiene was confirmed by presence of coliforms,* S. aureus,* and* Enterococcus* spp. The use of plastic covers, multipurpose sponges, and towels should be discouraged.

## 1. Introduction

Foodborne diseases remain a challenge globally, with higher incidence rate in developing countries. In 2010, the World Health Organization's Foodborne Disease Burden Epidemiology Reference Group estimated 582 million cases of foodborne diseases and 351 000 associated deaths worldwide [1]. Furthermore, elderly people, children aged less than 5 years, pregnant women, and individuals with low immune systems could be more vulnerable to foodborne diseases [2]. Every year, contaminated food contributes to 1.5 billion cases of diarrhoea in children, resulting in more than three million premature deaths worldwide [3].

Foodborne diseases originating from home have been increasingly reported recently and now considered to be an important aspect of public health [4, 5]. Households have been reported as the second most important venue for foodborne diseases after restaurants [6]. The incidence of home-based foodborne illnesses could be difficult to interpret due to various food sources and underreporting of illness [4, 6]. A number of factors could contribute to foodborne diseases in the home, including types of food supply, domestic activities taking place in the kitchen, hygienic practices, attitudes, belief, experience, and knowledge of every member of the household [4, 7, 8]. Experimental studies have concluded that cross-contamination of bacteria which could cause foodborne illnesses such as* S. aureus*,* Salmonella *spp., and* Campylobacter *spp. could occur from fleshy food to raw foods, kitchen surfaces, and equipment, including chopping boards and knives [4, 9, 10]. It has also been reported that 50% of foodborne diseases were due to inappropriate food storage and 28% were due to cross-contamination [11]. Poor hygiene was found to significantly affect the presence of* Escherichia coli* 0157:H7 in homemade hamburgers [12].

Bacteria responsible for foodborne disease could cause biofilm on food contact surfaces such as tables which could disseminate the potential pathogens continuously in the kitchen environment as well as ultimately affecting food quality and safety [13]. The bacterial appendages, fimbriae, flagella and surface polysaccharides have been extensively studied for their contributions to the formation of biofilms by* E. coli*,* Proteus* spp.,* Pseudomonas* spp.,* Klebsiella* spp., and* Salmonella* spp. [13].* Proteus* spp. have often been responsible for both food spoilage and food poisoning [14] whereas* Pseudomonas* spp., which are known to cause off-odours and off-flavours in food, have more often been cited as responsible for food deterioration and spoilage [15, 16].

In Mauritius, data from Ministry of Health and Quality of Health has indicated an ascending trend in the number of reported food poisoning cases, which was 2.0 cases in 2001 and increased to 31.0 cases per 100,000 midyear population in 2013. Furthermore, in 2013, diarrhoea and gastroenteritis of presumed infectious origin were the second cause of hospital discharge [17]. It would be impossible to estimate the percentage of home-based foodborne outbreaks, although it cannot be neglected. There is a need to study the sources and possible causes of foodborne diseases in household kitchens. Therefore, this study aimed to study the hygienic practices of a random sample of individuals in their home kitchens. The bacterial load and profile of potential pathogens and food spoilage bacteria from the home kitchen tables, dining and preparation tables, were investigated and compared. The various factors which might affect the load and presence of potential pathogens and food spoilage bacteria were also studied.

## 2. Material and Methods

### 2.1. Study Design

For the purpose of the study, a survey was initially carried out, followed by laboratory investigations. A questionnaire was designed which included four sections: firstly, general information of the family under study (age of members, family size/type, and diet); secondly, kitchen set-up (details of dining and preparation tables and their materials and cover and uses); thirdly, hygiene practices in the kitchen (hand washing frequency, use of chopping board); and, fourthly, food safety knowledge. The study was approved by the Department of Health Sciences, University of Mauritius.

### 2.2. Sample Collection

A total of 53 households provided the samples which were collected using sterile cotton swabs by swabbing over a 5 cm × 5 cm surface area of kitchen tables. From each kitchen, four samples were obtained, one from dining table in the morning, one from dining table in the afternoon, one from preparation table in the morning, and one from preparation table in the afternoon. All the 212 samples were processed within 24 hours.

### 2.3. Laboratory Investigations

All kitchen samples were processed for a bacterial count which was read as colony forming units (CFU), followed by the isolation and identification of potential pathogens. A serial dilution was carried out from the original sample and spread plate technique was done to determine the CFU/25 cm^2^. The samples were also streaked on sterile Nutrient Agar, MacConkey Agar, Bile Aesculin Agar, Salmonella Shigella Agar, Cetrimide Agar, and Sabouraud Agar (all from HiMedia, Mumbai, India). The potential pathogens were identified by conventional methods such as gram staining and biochemical tests such as catalase, coagulase, urease, oxidase, indole, methyl red, citrate, phenylpyruvic acid, and Kligler iron agar slant.

### 2.4. Statistical Analysis

Data analysis was done using SPSS v.19.0. Descriptive statistics were used to summarise demographic data. Independent sample *t*-test was used to calculate and compare between the bacterial load from the various sources. The odds ratio and difference in the prevalence of the potential pathogens were determined using Pearson's *χ* ^2^ test. A *p* value of less than 0.05 was read as significant.* Odds ratio* (OR) has been used to measure the association between potential pathogens and factors such as demographic details, types of table, usage of towels, and diet.

## 3. Results

### 3.1. Questionnaire

The demographic details of the families have been detailed on Table 1.

The kitchen was busiest during dinner time (45.3%) followed by morning breakfast (26.4%), lunch (17.0%), and afternoon tea time (11.3%). Of the 53 dining tables, 44 were made of wood and 9 were made of plastic material. None of the plastic dining tables were covered while 37 (84.1%) of the wood tables were covered with plastic cover with the rest covered with cloth material. A total of 37 preparation tables were made of ceramics and 16 were made of wood material.

It was noted that 17 (32.1%) households used their preparation tables as chopping boards and 27 (50.9%) used the same chopping board for both vegetables and fleshy foods. Only 21 (39.6%) of the respondents reported washing their hands always before preparing a meal or before eating. The frequency at which the kitchen was entirely cleaned was found to be daily for 18.9%, weekly for 58.8%, bimonthly for 17.0%, and monthly for 5.7%. For cleaning of the kitchen tables, 25 (47.2%) used multipurpose sponges, 13 (24.5%) used separate sponges, 13 (24.5%) used multipurpose kitchen towels, and 2 (3.8%) used separate kitchen towels. A high percentage of the respondents (96.2%) reported that food safety was very important.

### 3.2. Laboratory Investigations

Out of the 212 samples, 168 (79.2%) showed bacterial growth while yeast was noted in 27 (12.7%). The mean CFU/25 cm^2^ from the kitchen tables per day was 3264, with a higher prevalence from the preparation tables compared to the dining tables (3433 versus 3095), although the difference was not significant. The time of collection was not found to affect the CFU significantly.

The material of the tables was found to affect bacterial load. Dining and preparation tables made of plastic had higher CFU compared to those made of wood (*p* < 0.05). Furthermore, tables covered with plastic covers had higher CFU compared to cloth materials (*p* < 0.05). A significantly higher CFU/25 cm^2^ was noted from preparation tables which were also used as chopping boards (11185 versus 4839; *p* < 0.05).

Good hand washing practice, that is, always washing hands before preparing meals or eating, was significantly associated with lower CFU from both dining and preparation tables (*p* < 0.05). The tables cleaned with multipurpose sponges had the highest load with 8475 CFU/25 cm^2^ followed by multipurpose kitchen towels which had 6049 CFU/25 cm^2^, with separate sponges 3670 CFU/25 cm^2^ and separate kitchen towels 826 CFU/25 cm^2^. The difference was statistically significant.

The potential pathogens isolated from the samples have been detailed in Table 2.

A higher prevalence of coliform was noted from preparation tables compared to dining tables (28.3% versus 17.9%; *p* < 0.05; OR = 1.31 (1.01–1.73)), both in the morning (28.3% versus 19.9%; *p* < 0.05: OR = 1.28 (1.01–1.69)) and in the afternoon (28.3% versus 17.0%; *p* < 0.05: OR = 3.5 (1.02–1.77)).* Pseudomonas *spp. was also significantly more prevalent from the preparation table compared to dining tables (15.7% versus 5.1%; *p* < 0.05; OR = 1.53 (1.14–2.6)). Among samples collected from the preparation tables,* Enterococcus *spp. was more prevalent in the morning samples (45.3% versus 28.3%; *p* < 0.05; OR = 1.42 (1.02–2.06)).

A significant increase in the prevalence of coliform and* Enterococcus *spp. was found with increasing number of residents, children, adults, and elderly people (*p* < 0.05). It was also noted that more frequent cleaning of the kitchen and better hand hygiene, such as washing hands before preparing every meal or having meals, significantly decreased the prevalence of coliforms and* Enterococcus *spp. (*p* < 0.05). The other factors which significantly affected the presence of coliform have been detailed in Table 3.

*Enterococcus* was also isolated at higher prevalence from households on nonvegetarian diets (*p* < 0.05) and from preparation tables which were also used as chopping boards (56.4% versus 26.9%: *p* < 0.05: OR = 2.13 (1.30–3.51)).

*S. aureus* was more prevalent when the same chopping board was used for both vegetables and fleshy foods (22.2% versus 5.8%: *p* < 0.05: OR = 2.44 (1.87–3.19)). The association between potential pathogens and food spoilage bacteria from the kitchen tables and the cleaning materials used to clean the kitchens were also enquired (Table 4).

## 4. Discussion

It is now accepted that the prevalence of foodborne illnesses originating from home kitchens could not be neglected. However, most countries have not yet established adequate surveillance or reporting mechanisms to track home-based foodborne illnesses which could be due to technical and financial restraints.

In this study, it was found that although a very high percentage of respondents reported that food safety was a very important matter, only half of them used separate chopping board for vegetables and fleshy foods. Furthermore, only 39.6% adhered to good hand washing practice before handling food. It could be that either knowledge was not complete or knowledge was not always put into practice. Previous studies have also reported that knowledge and guidance in food safety do not always help in changing behavior [4].

The cleaning of the kitchens was done at different frequencies and more frequent cleaning was associated with lower prevalence of coliforms and* Enterococcus *spp. Food preparation and cleaning in the kitchen have been reported to be routine tasks [18] which could be mundane and taken for granted [19]. In a kitchen, the process of cleaning has been reported to vary from one household to another. Some people might clean to remove debris from the tables, some would tidy the surfaces, and very few would actually clean with the aim of removing microbes [4]. Therefore, microorganisms could very easily be transferred from one place to another.

The prevalence of* E. coli* and* Enterococcus* spp. was found to increase significantly in presence of elderly members and family size. It has been previously reported that food safety at home could be affected by the actions of every member using the kitchen [4]. Furthermore, the hands of older individuals were found to have a higher prevalence of coliforms compared to younger ones [7]. The elderly might be less strict about hygiene in the kitchen as they have been brought up in an era when processed food was consumed to lesser extent, refrigeration of foods was not in vogue, and the food supply chain was shorter [4].

In this study,* S. aureus* was the third most common potential pathogen isolated and was more prevalent when the same chopping board was used for both vegetables and fleshy foods. In an experimental study,* S. aureus* was found to have the highest rate of cross-contamination as compared to* Campylobacter*,* Salmonella,* and* E. coli* [10]. The presence of* S. aureus* on kitchen surfaces and food handlers hands has been associated with poor hygiene as the bacteria are highly susceptible to heat [7] and low concentrations of antibacterial dishwashing liquids [20].

As expected, a higher prevalence of potential pathogens was found from preparation tables compared to dining tables. The preparation tables are in contact with raw and fleshy foods more often. The presence of* S. aureus* and coliforms on kitchen counters and chopping boards has been previously reported. A significant increase of these potential pathogens was noted when the hands of the participants were positive to the same bacteria [7]. The use of preparation tables as chopping boards should be discouraged as this study found that such a practice significantly increased the CFU and prevalence of* Enterococcus*. One previous study reported an increase in prevalence of* S. aureus* and* E. coli* when preparation tables were used as chopping boards [7].

It was also revealed in this study that preparation tables made of wood have higher prevalence of coliform and* Enterococcus *spp. The nature of wood which is porous might allow penetration of juices from foods and bacteria, hence preventing their removal during cleaning and favouring their colonisation. Furthermore, plastic covers on both dining and preparation tables were associated with potential pathogens. The use of plastic covers on preparation tables should be discouraged as it was associated with high prevalence of coliforms. The cloth covers did not have coliform as the covers were most probably removed and washed as soon as they appear dirty whereas plastic covers might be wiped with sponges or towels to clean them for further use.

The hydrophobicity and roughness of surfaces together with the strain and surface physicochemical properties of the bacteria could affect initial adhesion process of foodborne bacteria to kitchen materials [9]. A review has concluded that strains of* Listeria monocytogenes* and* Salmonella enteritidis* could bind to various common surfaces in the kitchen including stainless steel, polypropylene, cutting board, and silestone, but with different degree of adhesion [9].* E. coli* and* S. aureus* survived on polyethylene materials for longer period of time [21]. The irregular surfaces of plastic material could favour the accumulation of organic matter and food residues, which could increase the attachment and survival of bacteria [22].

Several studies have concluded that kitchen cloths and sponges become contaminated during use and could be important in cross-contaminating kitchen utensils and surfaces [23, 24]. This study did not isolate bacteria directly from sponges. However, a higher mean CFU and potential pathogens were noted from kitchen tables which were cleaned with multipurpose sponges and towels compared to separate ones. Studies have concluded that* S. aureus* and other foodborne illness causing bacteria could be transmitted from contaminated sponges to kitchen surfaces [24]. Furthermore, it was reported that washing of sponges contaminated with food did not reduce the bacterial load significantly [20]. Therefore, the use of multipurpose sponges and towels should be avoided in kitchens.

The association of coliforms and* S. aureus* with foodborne diseases has been well documented.* Enterococcus* have also been recently studied as potential indicators of faecal contamination on hands as they are present in large numbers in human faeces and persist in the environment [25]. In the United Kingdom, enterococci are regarded as secondary indicators of faecal pollution [26]. The World Health Organization has recommended the adoption of enterococci as an indicator of recreational water quality [25, 27]. Faecal enterococci from human beings have been reported to be avirulent [25]. However, their presence on kitchen tables, towels, and sponges would indicate lack of hygiene which could eventually affect food safety.

## 5. Conclusion

The present study revealed that kitchen tables at home could be very important sources of potential pathogens which have been reported to cause foodborne illnesses. The use of plastic covers on kitchen tables, multipurpose sponges, and towels should be discouraged in the kitchen. Lack of hygiene was confirmed by presence of coliforms,* S. aureus,* and* Enterococcus* spp. on the tables. Furthermore, people should be encouraged to apply basic food hygiene practices at home to ensure food safety.

## Figures and Tables

**Table 1:** Demographic details of families.

Details	*N* (%)
Number of children	
None	17 (32.1)
1-2	27 (50.9)
3-4	9 (17.0)
Number of residents	
1-2	6 (11.3)
3-4	22 (41.5)
5-6	23 (43.4)
7-8	2 (3.8)
Number of adults	
1-2	16 (30.2)
3-4	34 (64.2)
>4	3 (5.7)
Number of elders	
None	30 (56.6)
1-2	23 (43.4)
Type of family	
Couple only	4 (7.5)
Nuclear	38 (71.7)
Extended	11 (20.8)
Diet of family	
Vegetarian	8 (15.1)
Nonvegetarian	45 (84.9)

*N*: sample size.

**Table 2:** Prevalence of potential pathogens and food spoilage bacteria isolated from the tables.

Microorganism	Prevalence of potential pathogen from tables						
	All (*n* = 212) %	Dining (*n* = 106) %	Preparation (*n* = 106) %	Dining AM (*n* = 53) %	Dining PM (*n* = 53) %	Preparation AM (*n* = 53) %	Preparation PM (*n* = 53) %
*S. aureus*	14.2	13.2	15.1	15.1	11.3	15.1	15.1
*Enterococcus *spp.	34.9	33.0	36.8	30.2	35.8	45.3	28.3
*Pseudomonas *spp.	10.4	5.7	15.1	3.8	7.5	15.1	15.1
*Proteus *spp.	3.8	1.9	5.7	0	3.8	3.8	7.5
Coliforms^#^	23.1	17.9	28.3	18.9	17.0	28.3	28.3

^#^ *E. coli* and *Klebsiella* spp. Dining AM: prevalence from samples collected in the morning from the dining tables. Dining PM: prevalence of potential pathogens from samples collected in the afternoon from the preparation tables.

**Table 3:** Factors affecting prevalence of coliforms.

Factors	Prevalence of coliforms
Family type	
Couple versus nuclear	0% vs 20.4%: *p* < 0.05: OR = 1.13 (1.06–1.20)
Couple versus extended	0% vs 40.9%: *p* < 0.05: OR = 1.62 (1.27–2.05)
Nuclear versus extended	20.4% vs 40.9%: *p* < 0.05: OR = 2.08 (1.25–3.45)
Dining table: covered versus uncovered	19.7% vs 0%: *p* < 0.05: OR = 1.76 (1.47–2.09)
Cover material of dining table: plastic versus cloth	31.0% vs 0%: *p* < 0.05: OR = 1.83 (1.43–2.33)
Material of preparation table: wood versus ceramic	54.5% vs 24.7%: *p* < 0.05: OR = 2.33 (1.34–4.06)
Preparation table: covered versus uncovered	65.0% vs 26.7%: *p* < 0.05: OR = 3.61 (1.58–8.25)

OR: odds ratio.

**Table 4:** Effect of usage of towels on the detection rate of potential pathogens and food spoilage bacteria.

Prevalence of	Cleaning materials of kitchen			
	Multipurpose sponge versus separate sponge	Multipurpose towel versus separate towel	Multipurpose towel versus separate sponge	Multipurpose sponge versus multipurpose towel
Coliforms^#^	32.0% vs 0%: *p* < 0.05: OR = 1.77 (1.51–2.06)	32.7% vs 0%: *p* < 0.05: OR = 1.23 (1.06–1.42)	32.7% vs 0%: *p* < 0.05: OR = 2.49 (1.92–3.21)	32.0 vs 32.7%: *p* = NS
*Enterococcus *spp.	59.0% vs 0%: *p* < 0.05: OR = 2.27 (1.80–2.85)	28.8% vs 0%: *p* < 0.05: OR = 1.12 (1.06–1.39)	28.8% vs 0%: *p* < 0.05: OR = 2.40 (1.88–3.08)	59.0% vs 28.8%: *p* < 0.05: OR = 1.52 (1.19–1.93)
*S. aureus*	11.5% vs 6.0%: *p* = NS	50.0% vs 6.0%: *p* < 0.05: OR = 9.8 (2.88–33.32)	50.0% vs 6.0%: *p* = NS	11.5% vs 50.0%: *p* = NS
*Pseudomonas *spp.	6.0% vs 0%: *p* < 0.05: OR = 1.55 (1.38–1.75)	30.8% vs 0%: *p* < 0.05: OR = 1.22 (1.06–1.40)	30.8% vs 0%: *p* < 0.05: OR = 2.44 (1.90–3.14)	6.0% vs 30.8%: *p* < 0.05: OR = 2.63 (1.80–3.83)
*Proteus *spp.	0% vs 0%: *p* = NS	15.4% vs 0%: *p* = NS	15.4% vs 0%: *p* < 0.05: OR = 2.18 (1.75–2.71)	0% vs 15.4%: *p* < 0.05: OR = 3.27 (2.56–4.19)

^#^ *E. coli *and *Klebsiella * spp. NS: nonsignificant.
